# Results of surgical treatment for Sprengle's deformity with vertical corrective scapular osteotomy

**DOI:** 10.1016/j.amsu.2020.03.006

**Published:** 2020-04-08

**Authors:** Mohammad Amin Aslani, Fateme Mirzaee, Amir Farahanchi Baradaran, Majid Eyvaz Ziaei, Zohreh Zafarani, Hamidreza Aslani

**Affiliations:** a Medical student of Tehran University of Medical Sciences, Knee and Sport Medicine Research Center, Milad hospital, Tehran, Iran; b MSc of Orthosis & Prosthesis, university of social welfare and rehabilitation sciences, Knee and Sport Medicine Research Center, Milad hospital, Tehran, Iran; c Shahid Beheshti University of Medical Sciences, Knee and Sport Medicine Research Center, Milad hospital, Tehran, Iran; d Orthopedic Surgeon, Knee and Sport Medicine Research Center, Milad hospital, Tehran, Iran; e Researcher in Knee and Sport Medicine Research and Education Center, Milad hospital, Tehran, Iran; f Orthopedic Surgeon, Knee and Sport Medicine Research and Education Center, Milad hospital, Tehran, Iran

**Keywords:** Sprengel's deformity, Surgical treatment, Vertical corrective scapular osteotomy, Clavicular osteotomy, Wake-up test

## Abstract

**Introduction:**

Sprengle deformity is the most common congential anomaly of shoulder complex that is the result of scapular placement in cephalad abnormal position. The purpose of this study is the evaluation of clinical and radiological results of vertical corrective scapular osteotomy and comparision of these results with previous studies.

**Methods:**

We retrospectively reviewed the results of the vertical corrective scapular osteotomy (VSO) with or without clavicular osteotomy and wake-up test in 31 consecutive patients at an average duration of follow up of 30 month (6 month-15 years). 22 patients were girls and 9 were boys. The average age of the patients was 7.3 years (3-13) at the time of surgery. We evaluated the clinical and radiological results of this method in last fallow-up. No funding was used for this study and there are no conflicts of interest.

**Results:**

31 surgical procedures were performed. All osteotomies were healed. No neurovascular complication. Postoperative the mean shoulder flexion and abduction were improved 30 and 36° respectively (p < 0.001). The mean improvement of superior scapular angle (S.S.A) and inferior scapular angle (I.S.A) were 16 and 21° respectively (p < 0.001).

**Conclusion:**

It is intuitive that more cosmetic scapular lowering with little chance of neurovascular problems can be achieved after VSO. In addition, scapular rotation can be corrected using this technique, which should be considered as one of the advantages of this technique.

We believe that a properly applied VSO procedure in severe deformities is safe with predictable outcomes in the treatment of a complex deformity that provides favorable functional and cosmetic outcomes in the longer term.

## Highlights

• Sprengle deformity is the most common congential anomaly of shoulder complex.
• Surgical options can vary from excision of the angle to some major procedures such as subtotal scapulectomy.
• Vertical scapular osteotomy (VSO) is considered a relocation procedure that can provide cosmetic and functional improvement.
• More cosmetic scapular lowering with little chance of neurovascular problems can be achieved after VSO.
• Scapular rotation can be corrected using this technique.

## 1 Introduction

**Fig. 1:**
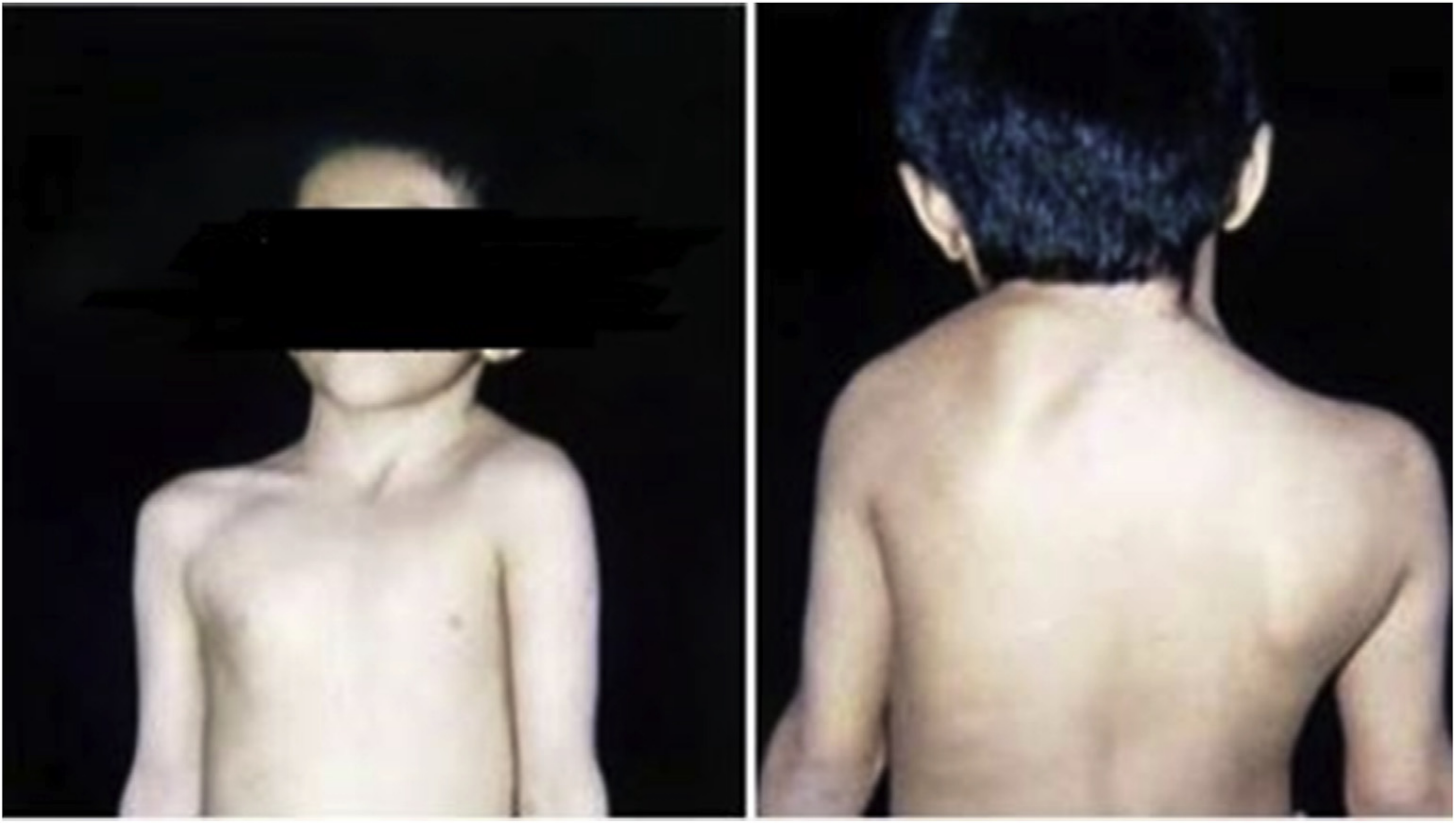
deformity of left shoulder in sprengle disease and. Scapular asymmetry.

Sprengel shoulder or congenital elevation of the scapula is associated with abnormal descending and altered position as well as the anatomy of the scapula [[1], [2], [3], [4]] [Fig. 1]. It is the most common congenital deformity of the shoulder girdle with female -to-male ratio of about 3:1 [5]. Shoulder abduction is mostly limited because of stiffed scapulothoracic articulation and an inferiorly rotated glenoid [6].

In about one-third of the patients, a pathologic factor might be found including a fibrous or a bony omovertebrale bridge between the cervical spine and the scapula [1].

Surgical options can vary from excision of the angle [7,8] to some major procedures such as subtotal scapulectomy. Vertical scapular osteotomy (VSO) is considered a relocation procedure that can provide both cosmetic and functional improvement [9,10]. This technique was first described by Konig in 1914, which was popularized by Wilkinson and Campbell [11]. The choice of surgery is still based on surgeon's preference [2,12,13].

In this study, we aim to assess the clinical and radiological results of vertical corrective scapular osteotomy with or without clavicular osteotomy and a wake-up test. This project has been reported in line with the SCARE criteria (14).

## 2 Methods

After receiving approval for the study, we conducted a retrospective case series review of the patients operated for Sprengle deformity at 2 major hospitals from 2002 to 2018. Thirty-one patients including 22 females and 9 males with unilateral involvement were included in this study**.** All patients had undergone vertical scapular osteotomy with or without wedge resection by 2 surgeons (MEZ and HRA). Patients were invited for a follow-up visit and improvement was assessed both clinically and radiologically. The mean age at the time of surgery was 7.5 years and the mean follow-up time was 30 months after the index surgery ranging from 6 months to 16 years.

Patients were assessed clinically before and after surgery and the deformity was graded according to the Cavendish classification [14] [Table 1]. Anteroposterior views of the shoulder were done in the standing position. We also asked the parents about functional outcome and cosmetic satisfaction.

**Table 1:** Cavendish's clinical classification.

Grade I	Very mild, shoulder level, and deformity invisible when dressed
Grade II	Mild, shoulders almost level, and lump visible in web of the neck when dressed
Grade III	Moderate, shoulder elevated 2–5 cm, and deformity easily visible
Grade IV	Severe, superior angle of scapula near occiput, with or without neck webbing

## 3 Operative technique

Surgical technique was based on the modification described by Wilkinson [11]. The patient was placed in the lateral position, and the upper half of the chest to the opposite side of the ribs, the entire neck, the entire upper limb, and the posterior aspect of the neck were fully prepped.

A vertical incision of about 2 cm lateral to the vertical border of the scapula was carried down. The periosteum overlying the scapula was reflected. Osteotomy was done with or without wedge resection at about 1 cm lateral to the vertebral border using an oscillating saw [15]. Offset holes were drilled into the scapula adjacent to the osteotomy line osteotomy. Any fibrous bands were palpated and divided. Fragments were aligned in the proper position and secured with non-absorbable sutures passing through the holes. The incision was closed in layers. The arm was supported in a sling after surgery for 1–2 weeks after which gentle active and passive motion started with progression to strengthening exercises.

**Fig. 2:**
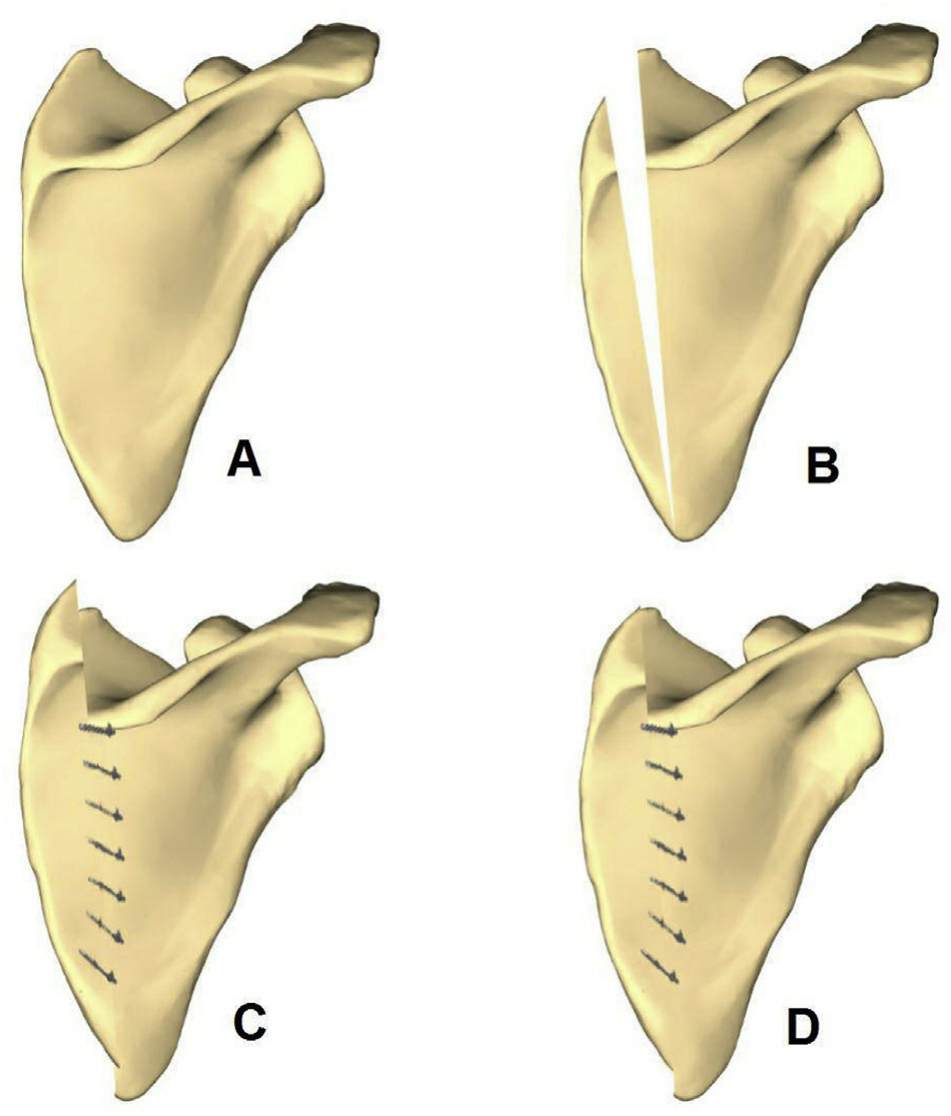
Changing in Wilkinson method and remove wedge shape bone.

To prevent scapular winging, we placed the lower corner of the scapula under the latissimus dorsi. In patients with highly rotated scapula, we removed a bone wedge off of the scapula. We removed the omovertebral in 10 patients extraperiosteally, and removed a bone wedge in 4 other to correct the scapular rotation [Fig. 2].

Clavicle was morcellized in a same session in patients older than six years, with higher grades (more than III or IV), and with existing omovertebral and restricted scapular movement to prevent damage the brachial plexus and to prevent compression of the neurovascular bundle against the ribs [16].

**Fig. 3:**
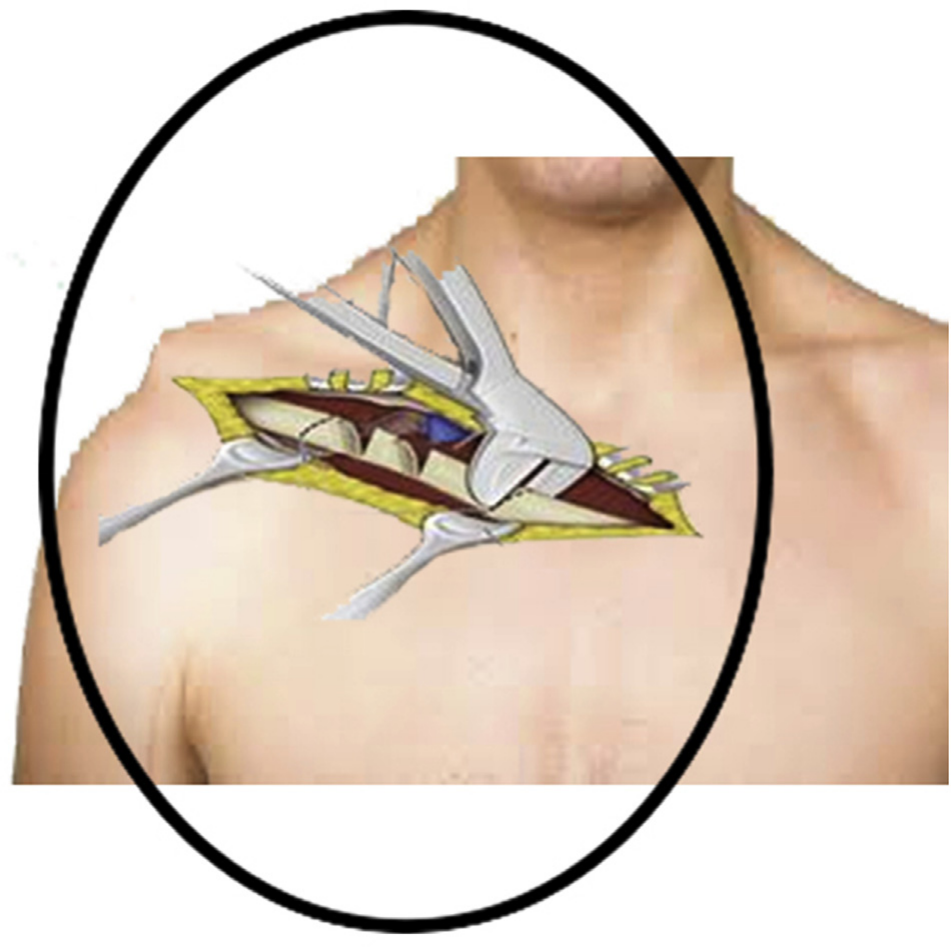
clavicular osteotomy.

For this stage, we first did the osteotomy of the clavicle. A supraclavicular curvilinear incision was made above the clavicle. After cutting the periosteum, the middle third of the clavicle was crushed into pieces and left in place. The periosteum, subcutaneous tissue, and skin were repaired in layers [Fig. 3]. After dressing the clavicular incision, the patient was positioned prone for scapular osteotomy.

Patients were given instruction about the wake-up test. It was done in all patients to prevent brachial plexus injury. The intraoperative wake-up test is still considered to be the gold standard, which is done by waking up the patients during spinal procedures [17,18]. This technique was performed successfully by Vauzella and Stagmara in 1973 for the first time [19]. Before surgery, the necessary training has to be given to the patients and their parents. It is important to explain the process and reassure the patients that this is a controlled event. This will minimize patient's anxiety and help ensure cooperation during the assessment. Patients should understand that they will be awakened briefly during the surgery and will be asked to follow the commands and to move their hand and elbow on the operating side. All of our patients received detailed instructions about this test several days before surgery. They were assured that they would feel no pain, and would be re-anesthetized quickly.

## 4 Results

The study included 9 male and 22 female patients with a mean age of 7 years at the time of surgery (range: 3 years–13 years). The mean follow-up time was 30 months. The deformity was in the left shoulder in 9 and right in 18 patients, while 4 had bilateral deformity. The most common accompanying anomaly was scoliosis in 14 patients. Omovertebral bone was found in 7 patients. Surgical scar hypertrophy was developed in 3 patients. One patient was suspicious for superficial infection, which was improved with antibiotic therapy. No brachial plexus lesion or scapular winging was observed in our patients. VSO alone was done in 12 patients while the other 19 underwent morcellization of the clavicle as well.

All patients improved functionally and cosmetically at the final follow-up. We used the grading system of Cavendish to evaluate the cosmetic improvement [10,15,20,21]. There was improved cosmetic with a decrease of at least 1 Cavendish Grade in all patients. The deformity was corrected completely in 8 patients. Of 31 patients at the final follow-up, 11 were rated as grade I, 9 as grade II, and 3 as grade III. The differences in pre- and post-operative shoulder flexion and abduction, scapular elevation, scapular rotation, and Cavendish score showed improvement in all patients [Table 2].

**Table 2:** Evaluation of the results of Cavendish scale, shoulder range of motion, scapular elevation and scapular rotation.

variables	Pre-operatively Mean (minimum–maximum)	Post-operatively Mean (minimum–maximum)
Cavendish scale	2.9 (2–4)	1.7 (0–3)
Shoulder abduction	123 (60–70)	159 (90–180)
Shoulder flexion	132 (70–170)	163 (110–180)
scapular elevation	2 cm (0.3–3.5)	0.5 cm (0–1.4).
S·S.A	30 (13–63)	46 (21–74)
I·S.A	48 (22–78)	28 (10–40).

Superior scapular angle (S·S.A) and inferior scapular angle (I·S.A).

**Fig. 4:**
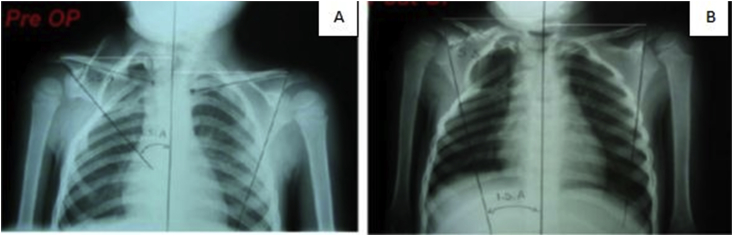
measurement method of upper and lower scapular angle in radiography. A. Preoperative B. Postoperative

To measure the rotation of the shoulder on anteroposterior radiography, a horizontal line was drawn between the center of the acromioclavicular joint and the center of the sternoclavicular joint, and a second line was drawn from the center of the acromioclavicular joint to the lower scapular angle tangential to the lateral border. The third line was drawn vertically in the middle of trunk connecting the spinous processes of the vertebras. We measured 2 angles that were created by these lines: 1. The upper scapular angle was between the first and the second line, which is 75° in normal people. 2. Lower scapular angle was between the second and the third line, which is 15° in normal people [Fig. 4].

After correcting the rotation, upper scapular angle was increased while decrease lower scapular angle was decreased to bring the medial border of the scapula in a more vertical position.

## 5 Discussion

Sprengel deformity is a congenital deformity associated with malposition and dysplasia of the scapula, which causes disfigurement and limitation of shoulder movement [22,23]. As already mentioned, several surgical methods have been defined for the surgical correction of Sprengel deformity [11,24,25]. The most frequently performed surgical procedures for correction of an abnormally elevated scapula are the techniques of Green, Woodward, and vertical scapular osteotomy. Green proposed excision of the omovertebral bone and supraspinatus part of the scapula with the release of the muscles off of the scapula, while Woodward suggested transferring the origin of the trapezius muscle over the vertebras to the lower levels. Although the techniques are quite different, both have shown good results [26].

We used vertical scapular osteotomy with or without clavicular osteotomy and wake-up test that is providing a decent visualization of the superomedial scapular angle and probably better improvement in shoulder abduction and flexion [10,15]. MacMurtry et al., in 2004 and Abdelaziz et al., in 2015 reported their experiences with this technique in 12 and 7 patients respectively [10,15]. They showed acceptable amount of correction of scapular elevation, rotation, range of motion (flexion and abduction) after VSO with improved Cavendish score.

In our study, improvement in abduction was more in patients with omovertebral bone. In our study, 23% of the patients had omovertebral bone, which has been reported between 18% and 60% in the other studies [11,14]. It is recommended to remove it completely if it is not part of the vertebral column [3,27].

After osteotomy, release of the soft tissue around the scapula, and removing a wedge-shaped bone, we were able to bring the scapula down and to correct the rotation. In this technique, there is a minimal dissection with small incision, scar is more cosmetic, and bleeding is less than the other techniques. Moreover, we did not find any postoperative winging of scapula in our patients.

Brachial plexus injury is the most important complication reported in the literature [10,11,24,26,28], however, this did not happen in any of our patients even in older patients. This is assumed to be prevented after using wake-up test in all patients and morcellation of clavicle in higher risk patients.

Some parts of the scapula are ossified at birth, but the glenoid cavity, coracoid process, acromion, vertebral border, and the inferior angle are cartilaginous. Ossification occurs in the middle of the coracoid process between 15th and 18th month after birth, and the remaining parts ossify between 14th and 20th years of life [29,30]. Hypothetically, scapular osteotomy and displacement of the more lateral portion inferiorly allows the secondary bone core to extend more inferiorly to form a scapula more comparable to the other side, which compensates some of the scapular deficiency. This was also observed on follow-up radiographies; however, future studies are required to prove it. We believe that the most favorable cosmetic and functional results in Sprengel deformity can be obtained after VSO combined with morcellation of clavicle and wake-up test, which has low morbidity. The wake-up-test, also known as Stagnara's test [19] is a reliable and practical method for early detection of any neurological problems during a major spinal surgery with very few reported complications [31].

## 6 Conclusion

It is intuitive that more cosmetic scapular lowering with little chance of neurovascular problems can be achieved after VSO. In addition, scapular rotation can be corrected using this technique, which should be considered as one of the advantages of this technique.

We believe that a properly applied VSO procedure in severe deformities is safe with predictable outcomes in the treatment of a complex deformity that provides favorable functional and cosmetic outcomes in the longer term.

## Ethical approval

The ethical approval for the publication of this retrospective review was exempted by our institution because all of the data were collected from clinical records and imaging systems for routine perioperative planning.

## Sources of funding

This research did not receive any specific grant from funding agencies in the public, commercial, or not-for-profit sectors.

## Author contribution

ME.Z: Therapist.

F.M: Writer & data analysis.

Z.Z & MA.A & A.FB: Editing the manuscript.

H.A: Editing the manuscript & Corresponding author.

## Research registration number

Study registered with Iranian Registry of Clinical Trials 44045**.**

## Guarantor

Hamidreza Aslani.

## Consent

Written informed consent was obtained from all of the patient's fathers as they are minors, for publication of this case series and accompanying images. A copy of the written consent is available for review by the Editor-in-Chief of this journal on request.

## Provenance and peer review

Not commissioned, externally peer reviewed.

## Declaration of competing interest

The authors declare no conflicts of interest. The authors have no financial, consultative, institutional and other relationships that might lead to bias or conflict of interest.
